# Assessing a 6-h endpoint observation time in the lethality neutralization assay used to evaluate the preclinical efficacy of snake antivenoms

**DOI:** 10.1016/j.toxcx.2021.100087

**Published:** 2021-11

**Authors:** Gina Durán, Gabriela Solano, Aarón Gómez, Daniel Cordero, Adriana Sánchez, Mauren Villalta, Melvin Sánchez, Cecilia Díaz, José María Gutiérrez, Guillermo León

**Affiliations:** Instituto Clodomiro Picado, Facultad de Microbiología, Universidad de Costa Rica, San José, Costa Rica

**Keywords:** Antivenom potency, Lethality neutralization assay, Median effective dose, Median lethal dose, Replacement, Reduction, And refinement principle, Snake antivenom

## Abstract

The lethality neutralization assay in mice is the gold standard for the evaluation of the preclinical efficacy and specification fulfillment of snake antivenoms. However, owing to the animal suffering involved, this assay is a candidate to be replaced by *in vitro* alternatives or, at least, improved by the reduction of the number of animals used per experiment, the introduction of analgesia, and the refinement of the test. Since these tests are usually run for 24 or 48 h, one possibility to refine it is to shorten the endpoint observation time of the assay and so limiting the duration of suffering. To assess the effect of this modification of the standard procedure on the analytical properties of the assay, we compared the median lethal dose (LD_50_) and median effective dose (ED_50_) values, estimated through observation times of 6, 24 and 48 h. We used African and Latin American snake venoms and several batches of two polyspecific antivenoms. A significant correlation was found between LD_50_ and ED_50_ values estimated at the three observation times. Although some LD_50_ and ED_50_ values were significantly different at these time points, results of 6 h were robust enough to be used in the characterization of new antivenoms, the verification of specification compliance, and the parallel comparison of formulations. Our observations support the modification of the standard procedures used for assessing neutralizing ability of antivenoms by carrying out the observations at 6 h instead of 24 or 48 h, with the consequent reduction in the suffering inflicted upon mice during these assays. However, the shortening of the observation time in the lethality tests must be validated for each venom and antivenom before its introduction in the routine procedures.

## Highlights

• The lethality neutralization assay in mice is the gold standard for the preclinical evaluation of snake antivenoms.
• This assay is a candidate to be improved by the 3Rs philosophy (i.e., replacement, reduction and refinement).
• The effect of the reduction of the endpoint observation time on the analytical properties of the test was assessed.
• A significant correlation was found between LD_50_ and ED_50_ values estimated at 6 h and 24 or 48 h.
• A 6-h observation time limits the duration of the suffering without reducing the analytical quality of the test.

## Graphical abstract

**Figure:**
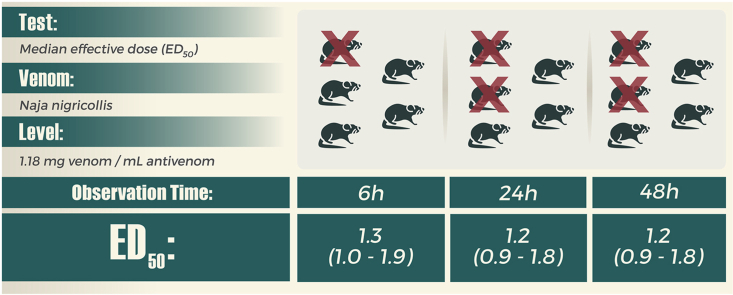

## 1 Introduction

The lethality neutralization assay in mice is the gold standard for the evaluation of the preclinical efficacy and specification fulfillment of snake antivenoms (WHO, 2016). In the standard procedure, this assay is carried out by mixing a constant challenge dose of venom with different dilutions of antivenom. Then, the mixtures are injected in groups of mice to determine the residual lethal activity of the non-neutralized venom. To ensure that all deaths induced by the venom are recorded, the protocols of the World Health Organization (WHO) indicate final observation times of 24 h for intravenous (IV) injections, or 48 h for intraperitoneal (IP) injections (WHO, 2016). Then, the recorded deaths are analyzed by Probits, Spearman-Karber or non-linear regression procedures to calculate the median effective dose (ED_50_) and the corresponding antivenom potency (Araujo et al., 2008; WHO, 2016).

Since the action of many snake venoms is based on the combined effects of several toxins (Chacón et al., 2015; Segura et al., 2017), the pathophysiological mechanisms of envenoming in mice are difficult to be modeled *in vitro*. Therefore, tests involving the use of mice, especially regarding the lethality assay, are still considered necessary to properly assess venom toxicity and preclinical antivenom efficacy (WHO, 2016), despite uncertainties about whether the mode of death in envenomed mice reflects that in human snakebite victims. However, in accordance with the principles of replacement, reduction and refinement (i.e., the 3Rs principles) in animal testing, efforts are being carried out in order to (a) replace the mouse lethality assay by properly validated *in vitro* alternatives, (b) reduce the number of mice used in the assays, and (c) refine the assay by the use of analgesia (Gutiérrez et al., 2021).

Reduction of the number of mice used per test can be achieved by modifying the experimental protocols based on the analytical properties of the assay (Solano et al., 2010). On the other hand, refinement of the method could be achieved by using routine analgesia to reduce pain experienced by mice during the test (Chacón et al., 2015; Herrera et al., 2018). Additionally, the shortening of the observation time and the application of euthanasia on animals, as soon as they show signs of severe envenoming, have been used as a refined modification of the classical protocol for toxicity assays (Barber et al., 2014). Although this modification reduces the pain, suffering and distress experienced by the animals, its impact on the analytical properties of the standard assays to test snake venom toxicity and antivenom neutralizing ability, has not been evaluated.

In this work, we determined the effect of the observation time on the estimation of the median lethal dose (LD_50_) values of some African and Latin American snake venoms, and on the ED_50_ values of several batches of two polyspecific antivenoms manufactured in Costa Rica against these venoms (i.e., EchiTAb-ICP and PoliVal-ICP), and evaluated the analytical suitability of the results obtained in assays performed using a 6 h observation time.

## 2 Materials and methods

### 2.1 Ethics

All procedures used in this study were approved by the Institutional Committee for the Care and Use of Laboratory Animals (CICUA) of Universidad de Costa Rica (Proceedings 82–08 and 39–20) and meet the International Guiding Principles for Biomedical Research Involving Animals (CIOMS, 1986). Most experiments carried out in this study corresponded to the routine quality control assays of antivenoms at Instituto Clodomiro Picado (ICP).

### 2.2 Venoms

Venoms of *Bitis arietans arietans*, *Echis ocellatus* and *Naja nigricollis* were purchased from Latoxan (Portes-dès Valence, France). Venoms of *Bothrops asper*, *Crotalus durissus pifanorum*, *Crotalus simus* and *Lachesis stenophrys* were collected from adult specimens maintained in captivity at the Serpentarium of Instituto Clodomiro Picado (ICP, University of Costa Rica, Costa Rica). After collection, venoms were stabilized by lyophilization and stored at −40 °C. Solutions of venoms were prepared immediately before use.

### 2.3 Snake antivenoms

Different batches of EchiTAb-ICP and PoliVal-ICP antivenoms, manufactured at ICP, were used. These antivenoms are polyspecific formulations constituted by whole immunoglobulin G (IgG) molecules purified by caprylic acid precipitation (Rojas et al., 1994) from the plasma of horses immunized with venoms of *B. a. arietans*, *E. ocellatus* and *N. nigricollis* (in the case of EchiTab-ICP; Gutiérrez et al., 2005), or *B. asper*, *C. d. pifanorum*, *C. simus* and *L. stenophrys* (in the case of PoliVal-ICP; Alfaro-Chinchilla et al., 2021).

### 2.4 Determination of median lethal dose (LD_50_)

Groups of five CD-1 mice were pretreated with the analgesic tramadol, administered by the subcutaneous (SC) route, at a dose of 50 mg/kg (Chacón et al., 2015) and then injected with different amounts of venom (dilution factor of 1.3) dissolved in 0.12 M NaCl, 0.04 M phosphate buffer, pH 7.2 (PBS). Volume of injection was 0.2 mL for IV route (in the case of the African venoms), or 0.5 mL for IP route (in the case of the Latin American venoms). The number of deaths was recorded at 6, 24 and 48 h. The LD_50_ and the corresponding 95% Confidence Interval (95% CI) were calculated by Probits (Finney, 1971).

### 2.5 Determination of median effective dose (ED_50_)

Groups of five CD-1 mice were pretreated with tramadol, administered by the SC route, at a dose of 50 mg/kg, and then injected with mixtures containing a challenge dose of venom and variable dilutions of antivenom (dilution factor of 1.5). Challenge doses were 3 LD_50_s for the venom of *N. nigricollis*; 4 LD_50_s for the venoms of *B. asper*, *C. d. pifanorum* and *L. stenophrys*; and 5 LD_50_s for the venoms of *B. arietans* and *E. ocellatus*. These challenge doses are the ones routinely used in the quality control of antivenoms at ICP. In the case of *C. simus*, 4, 5, 6, 7 and 8 LD_50_s were used as challenge dose to assess the effect of varying challenge doses on the results. PBS was used as solvent. The mixtures were incubated at 37 °C during 30 min before injection. Volume of injection was 0.2 mL for IV route (in the case of the African venoms and EchiTAb-ICP), or 0.5 mL for IP route (in the case of the Latin American venoms and PoliVal-ICP). The number of deaths was recorded at 6, 24 and 48 h. The ED_50_ and the corresponding 95% CI were calculated by Probits (Finney, 1971).

### 2.6 Statistical analyses

Differences in LD_50_ and ED_50_ values calculated at different observation times were evaluated by the overlapping of the 95% CI. In the case of ED_50_ values, a general linear model of repeated measures was also carried out along with their paired sample correlations. Normality, homogeneity of variances, and sphericity assumptions were tested, and the results of the general linear model were corrected by the Greenhouse-Geisser factor if the assumptions were not met. Values of p < 0.05 were considered as significantly different. Pearson's bivariate correlations were performed to compare the ED_50_ values calculated with deaths recorded at 6, 24 and 48 h. The tests were conducted using the IBM SPSS Statistics v25 software.

## 3 Results and discussion

### 3.1 Effect of the observation time on the LD_50_ value

After venom injection, the toxins distribute into the mouse body and induce systemic pathophysiological alterations which may lead to death. Time of death was shorter in venoms injected by the IV route (i.e., *B. arietans*, *E. ocellatus* and *N. nigricollis*) than in those injected by the IP route (*B. asper*, *C. d. pifanorum*, *C. simus* and *L. stenophrys*). This finding is likely due to the fact that IV route has a higher bioavailability and faster systemic distribution of venom than the IP route (Sanhajariya et al., 2018).

**Fig. 1:**
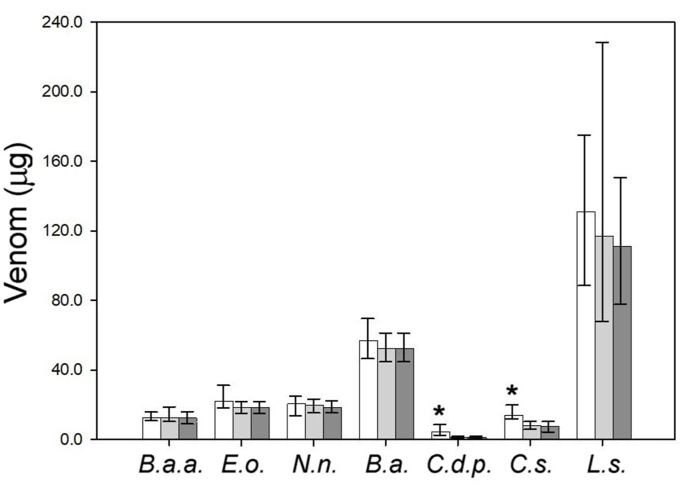
LD_50_ and 95% CI of several African and Latin-American snake venoms. *B.a.a*. (*B. a. arietans*), *E.o.* (*E. ocellatus*), *N.n.* (*N. nigricollis*), *B.a.* (*B. asper*), *C.d.p.* (*C. d. pifanorum*), *C.s.* (*C. simus*) and *L.s.* (*L. stenophrys*). Values correspond to observation times of 6 h (white bars), 24 h (light gray bars) and 48 h (dark gray bars). *Values obtained at 6 h were significantly different from the corresponding values obtained at 24 or 48 h.

For all venoms, as expected, the number of deaths increased dose-dependently. In the case of venoms of *B. arietans*, *E. ocellatus*, *N. nigricollis*, *B. asper* and *L. stenophrys*, most deaths occurred within 6 h of venom injection. Consequently, differences between LD_50_ values obtained at 6 h and those obtained at 24 h or 48 h, were lower than 22% and the 95% CI calculated at the three observation times overlapped (Fig. 1). These differences, which are characteristic of biological assays, were not significant.

In the cases of *C. d. pifanorum* and *C. simus*, the 95% CI calculated at 6 h did not overlap with those corresponding to 24 or 48 h (Fig. 1). Consequently, in these cases, an observation time of 6 h resulted in significantly higher LD_50_ values, making these venoms appear to be less toxic than when observations were done at 24 or 48 h. However, this does not reduce the validity of the results, as long as the experimental conditions (i.e., injection route, challenge dose and observation time) are considered when comparing and interpreting the data. Our observations support the modification of the LD_50_ assay protocol to reduce the observation time to a period of 6 h.

### 3.2 Effect of the observation time on the ED_50_ value

**Fig. 2:**
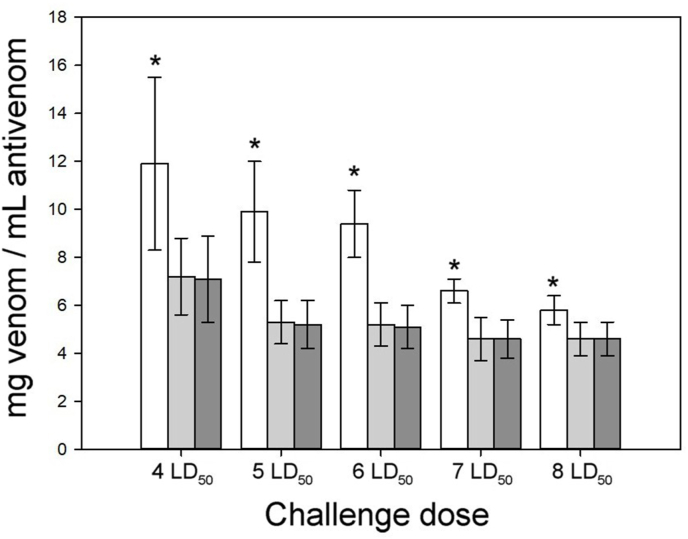
ED_50_ and 95% CI of PoliVal-ICP towards venom of *C. simus*, calculated with different challenge doses. Values correspond to observation times of 6 h (white bars), 24 h (light gray bars) and 48 h (dark gray bars). Values are presented as mean and SD of ED_50_ values of 5 different batches of PoliVal-ICP. *Values obtained at 6 h were significantly different from the corresponding values obtained at 24 or 48 h (4 LD_50_: F = 5.922_(2;12)_, p = 0.016; 5 LD_50_: F = 16.962_(2;12)_, p < 0.0001; 6 LD_50_: F = 25.889_(2;12)_, p < 0.0001; 7 LD_50_: F = 11.338_(2;12)_, p = 0.002; 8 LD_50_: F = 4.795_(2;12)_, p = 0.029).

The time lapse between injection of venom/antivenom mixtures and the time of death varied depending on the specific mixture. But for the same venom/antivenom combination, the time of death tended to be shorter as the venom/antivenom ratios increased. Moreover, as demonstrated by the comparison of ED_50_ values of PoliVal-ICP when using different challenge doses of *C. simus* venom, the time of death was shorter as the venom challenge dose increased, even at the same venom/antivenom ratio. On this basis, by selecting an appropriate challenge dose of venom, the differences in the number of deaths recorded at 6 h versus 24 or 48 h could be reduced (Fig. 2).

**Fig. 3:**
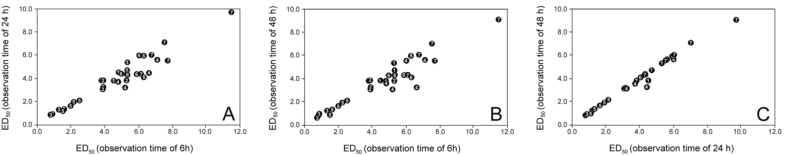
Pearson's bivariate correlations between ED_50_ values obtained at 6 and 24 h (A; r = 0.963; p < 0.0001), 6 and 48 h (B; r = 0.950; p < 0.0001), and 24 and 48 h (C; r = 0.992; p < 0.0001). Dots correspond to ED_50_ values of five batches of EchiTAb-ICP toward venoms of *B. a. arietans* (1), *E. ocellatus* (2) and *N. nigricollis* (3); and five batches of PoliVal-ICP toward venoms of *B. asper* (4), *C. d. pifanorum* (5), *C. simus* (6) and *L. stenophrys* (7).

When using challenge doses of 3 LD_50_s for the venom of *N. nigricollis*, 4 LD_50_s for the venoms of *B. asper*, *C. d. pifanorum* and *L. stenophrys*, 5 LD_50_s for the venoms of *B. arietans* and *E. ocellatus*, and 8 LD_50_s for the venom of *C. simus*, we found a significant correlation between ED_50_ values obtained with observation times of 6 and 24 h (r = 0.963; p < 0.0001; Fig. 3A); 6 and 48 h (r = 0.950; p < 0.0001; Fig. 3B); and 24 and 48 h (r = 0.992; p < 0.0001; Fig. 3C). This indicates that ED_50_ values obtained at a 6 h observation time could be used to express the neutralizing ability of antivenoms.

**Fig. 4:**
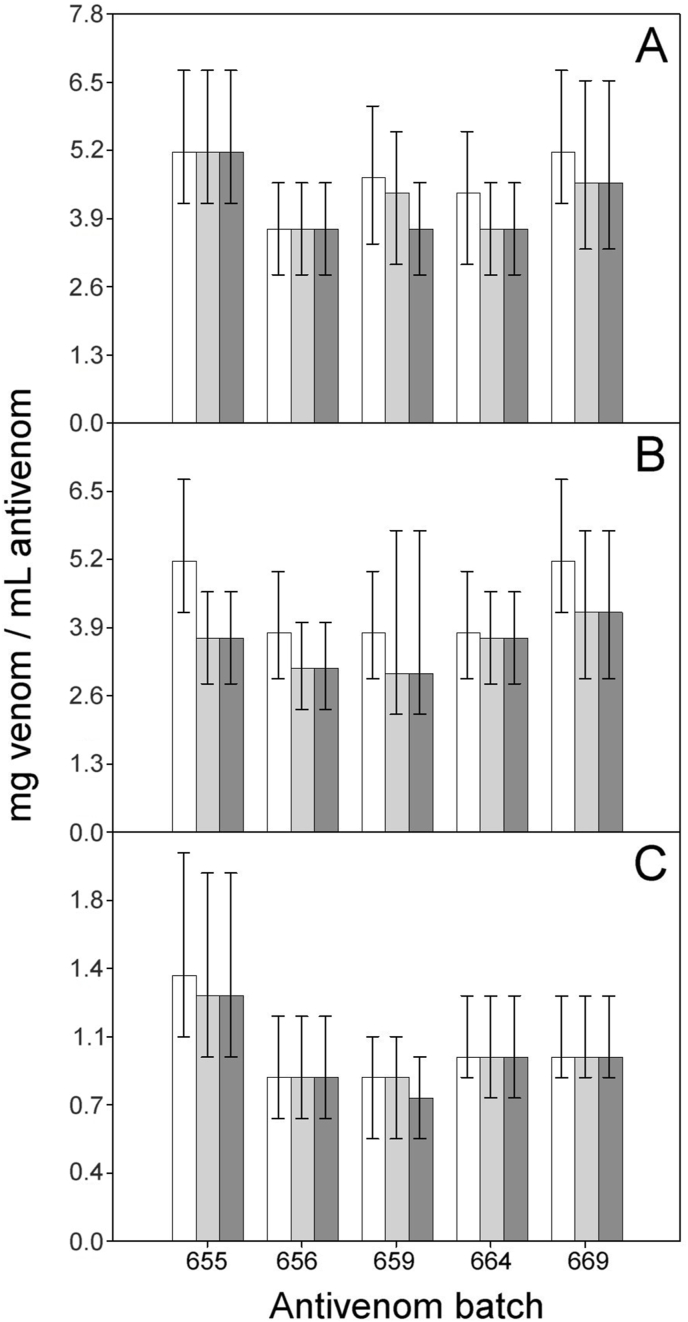
ED_50_ and 95% CI of five batches of EchiTAb-ICP toward the venoms of *B. a. arietans* (A), *E. ocellatus* (B) and *N. nigricollis* (C). Values were calculated at observation times of 6 h (white bars), 24 h (light gray bars) and 48 h (dark gray bars). No values obtained at 6 h were significantly different than the corresponding values obtained at 24 or 48 h (*B*. *a*. *arietans* F = 0.653_(2;12)_, p = 0.538; *E*. *ocellatus* F = 3.310_(2;12)_, p = 0.081; *N*. *nigricollis* F = 0.057_(2;12)_, p = 0.945).

**Fig. 5:**
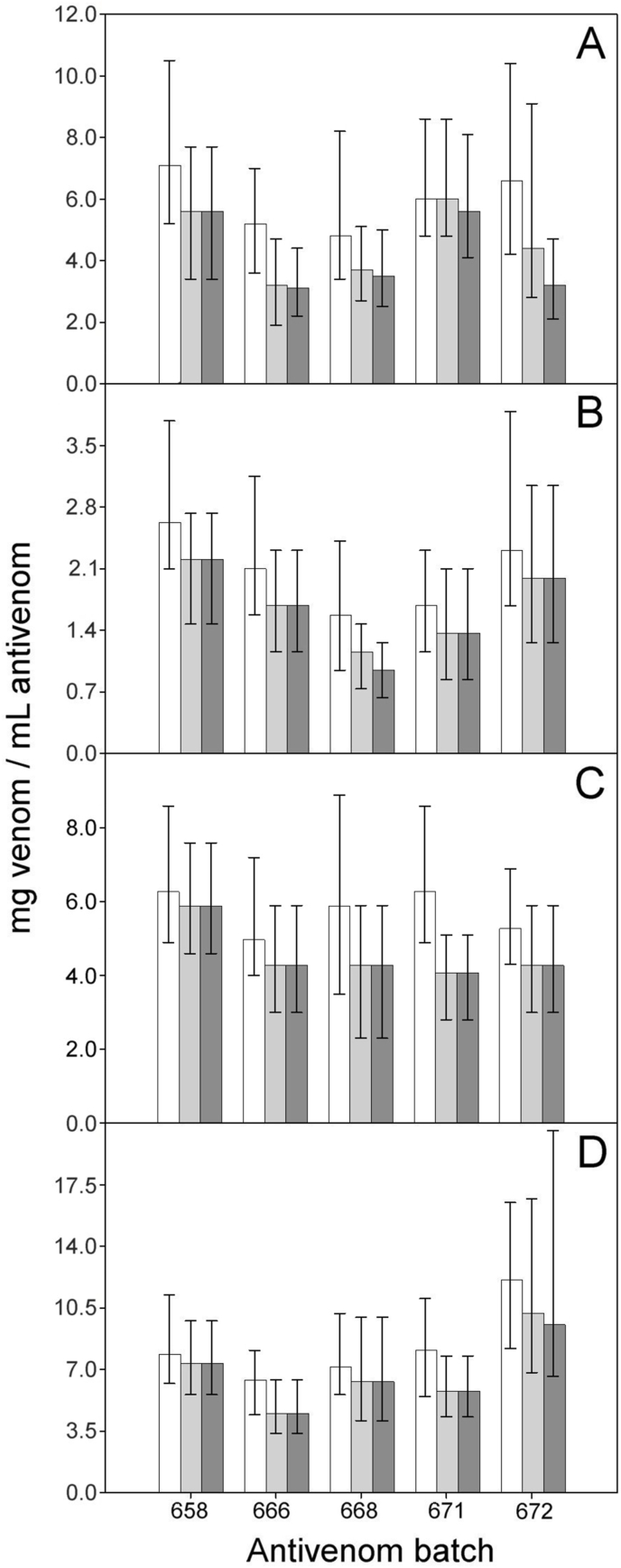
ED_50_ and 95% CI of five batches of PoliVal-ICP toward the venoms of *B. asper* (A), *C. d. pifanorum* (B), *C. simus* (C) and *L. stenophrys* (D). Values were calculated at observation times of 6 h (white bars), 24 h (light gray bars) and 48 h (dark gray bars). With exception of *C. simus* venom, (F = 4.795_(2;12)_, p = 0.029), no values obtained at 6 h were significantly different than the corresponding values obtained at 24 or 48 h (*B*. *asper* F = 3.135_(2;12)_, p = 0.080; *C*. *d*. *pifanorum* F = 1.275_(2;12)_, p = 0.315; *L*. *stenophrys* F = 0.932_(2;12)_, p = 0.420).

For all the venoms and antivenom batches tested, 95% CI calculated with deaths recorded at 6 h overlapped with the corresponding values calculated with deaths recorded at 24 or 48 h (Fig. 4, Fig. 5). This finding indicates that differences in ED_50_ values estimated at these three-time intervals are not significantly different. However, since the wide range of the confidence intervals could mask differences in neutralization, we evaluated the effect of the observation time on the ED_50_ values by a linear model of repeated measures.

For all venoms, 48–89% of the deaths occurred during the period of 6 h after injection of venom/antivenom mixtures, 88–100% in the first 24 h, and 100% at 48 h. The shortening of the observation time to 6 h resulted in an increment of the sensitivity (i.e., the ability to detect all mice rescued by the antivenom), and a decrease in the specificity (i.e., the ability to discriminate the survival caused by factors other than the antivenom) of the method. Consequently, when an observation time of 6 h was used, antivenoms tended to appear more effective (i.e., had higher ED_50_ values) than when the observation was done at 24 or 48 h. However, with the exception of *C. simus* venom, (F = 4.795_(2;12)_, p = 0.029), no value obtained at 6 h was significantly different than the corresponding values obtained at 24 or 48 h (Fig. 4, Fig. 5).

Similar effects in the numeric representation of ED_50_ values were previously described by other factors such as the way by which the mixtures of venom and antivenom are injected, and the venom challenge dose used (Solano et al., 2010). Consequently, the same neutralizing ability of an antivenom can be represented by different ED_50_ values, depending on the design of the methodology used to determine it. For example, the ED_50_ specification of PoliVal-ICP toward venom of *B. asper* is 3.0 mg/mL when tested by the IP route, using a challenge dose of 4 LD_50_s and an observation time of 48 h. On the other hand, the same specification is 6.0 mg/mL when tested by the IP route, using a challenge dose of 3 LD_50_s and an observation time of 48 h; or 7.0 mg/mL when tested by the IV route, using a challenge dose of 4 LD_50_s and an observation time of 48 h (Solano et al., 2010).

It is therefore suggested that, based on comparisons between the results of ED_50_ estimated by using 6, 24 and 48 h observation times, quality control laboratories and regulatory agencies can determine the specifications of the accepted values of ED_50_s of antivenoms when observation times are reduced to 6 h. In the above example for PoliVal-ICP, if observations are done at 6 h, the accepted ED_50_ value for this antivenom against *B. asper* venom would be 4.5 mg/mL instead of 3.0 mg/mL, which is the currently accepted value in our laboratory when determinations are done at 48 h. Each laboratory and regulatory agency should define the specifications for ED_50_ values at 6 h depending on the venom/antivenom being tested, by establishing correlations with values obtained at 24 or 48 h, following appropriate validation procedures.

The procedures used for the evaluation of the preclinical efficacy of antivenoms are subject of improvement by the reduction of pain, suffering and distress experienced by the animals. This is valid not only for antivenoms towards snake venoms, but also towards venoms of other venomous organisms such as scorpions, spiders and selected marine animals. In any case, the effect of these modifications on the analytical properties of the standard assays must be validated for each venom and antivenom system.

## 4 Conclusions

The procedures routinely used for the estimation of venom toxicity and antivenom potency, based on the mouse lethality assay, are of concern owing to the inherent suffering induced in mice during the tests. Therefore, efforts are being promoted in order to refine the protocols of these assays. The use of the analgesic tramadol has been previously used in the mouse lethality test (Chacón et al., 2015) and shown to be effective at a dose of 50 mg/kg (Herrera et al., 2018). In the present study we assessed whether it is feasible to reduce the observation time to 6 h in the estimation of venom LD_50_ and antivenom ED_50_. Results show that, LD_50_ and ED_50_ obtained at 6 h can be used to estimate, respectively, the toxicity of venoms and the neutralizing ability of antivenoms to characterize new formulations, verify compliance with specifications of batches serially produced at industrial scale, and compare two formulations analyzed in parallel. This modification in the protocol will demand a reconsideration of the accepted ED_50_ values for antivenoms, based on the comparison between results obtained at 6 h versus 24 or 48 h. Our observations provide support for a modification of the gold standard procedure used for assessing venom toxicity and antivenom potency by carrying out the observations at 6 h, with the consequent reduction in the suffering inflicted upon mice, according to the postulates of the 3Rs principles. However, the shortening of the observation time in the lethality assays must be validated for each venom and antivenom before its introduction in the routine procedures. This is particularly important in the case of venoms of species which have different mechanisms of toxicity. This task demands renewed studies by research groups and quality control laboratories.

## Credit author statement

Gina Durán: Conceptualizacion, Investigation, Formal analysis; Gabriela Solano: Formal analysis, Writing – review & editing; Aarón Gómez: Resources, Formal analysis, Writing – review & editing; Daniel Cordero: Writing – review & editing; Adriana Sánchez: Writing – review & editing; Mauren Villalta: Writing – review & editing; Melvin Sánchez: Resources; Cecilia Díaz: Writing – review & editing, Supervision; José María Gutiérrez: Conceptualizacion, Funding acquisition, Writing – original draft; Guillermo León: Conceptualizacion, Funding acquisition, Writing – original draft.

## Ethical statement

This manuscript presents an experimental study performed following the standard procedure of scientific ethics, including the use and care of experimental animals. All procedures used in this study were approved by the Institutional Committee for the Care and Use of Laboratory Animals (CICUA) of Universidad de Costa Rica (Proceedings 82–08 and 39–20) and meet the International Guiding Principles for Biomedical Research Involving Animals (CIOMS, 1986). Most experiments carried out in this study corresponded to the routine quality control assays of antivenoms at Instituto Clodomiro Picado (ICP).

## Declaration of competing interest

The authors declare the following financial interests/personal relationships which may be considered as potential competing interests:

The authors declare that they have no conflicts of interest regarding this study
